# Cancer mortality distribution in South Africa, 1997–2016

**DOI:** 10.3389/fepid.2023.1094271

**Published:** 2023-06-19

**Authors:** Mandlakayise Lucky Nhleko, Ijeoma Edoka, Eustasius Musenge

**Affiliations:** ^1^ Division of Epidemiology and Biostatistics, School of Public Health, Faculty of Health Sciences, University of the Witwatersrand, Johannesburg, South Africa; ^2^ Health Economics and Epidemiology Research Office, Department of Internal Medicine, School of Clinical Medicine, Faculty of Health Sciences, University of the Witwatersrand, Johannesburg, South Africa; ^3^ School of Public Health, Faculty of Health Sciences, University of the Witwatersrand, Johannesburg, South Africa

**Keywords:** cancer, mortality distribution, proportionate mortality ratios, causes of death, Africa

## Abstract

**Introduction:**

The mortality data in South Africa (SA) have not been widely used to estimate the patterns of deaths attributed to cancer over a spectrum of relevant subgroups. There is no research in SA providing patterns and atlases of cancer deaths in age and sex groups per district per year. This study presents age-sex-specific geographical patterns of cancer mortality at the district level in SA and their temporal evolutions from 1997 to 2016.

**Methods:**

Individual mortality level data provided by Statistics South Africa were grouped by three age groups (0–14, 15–64, and 65+), sex (male and female), and aggregated at each of the 52 districts. The proportionate mortality ratios (PMRs) for cancer were calculated per 100 residents. The atlases showing the distribution of cancer mortality were plotted using ArcGIS. Spatial analyses were conducted through Moran's I test.

**Results:**

There was an increase in PMRs for cancer in the age groups 15–64 and 65+ years from 2006 to 2016. Ranges were 2.83 (95% CI: 2.77–2.89) −4.16 (95% CI: 4.08–4.24) among men aged 15–64 years and 2.99 (95% CI: 2.93–3.06) −5.19 (95% CI: 5.09–5.28) among women in this age group. The PMRs in men and women aged 65+ years were 2.47 (95% CI: 2.42–2.53) −4.06 (95% CI: 3.98–4.14), and 2.33 (95% CI: 2.27–2.38) −4.19 (95% CI: 4.11–4.28). There were considerable geographical variations and similarities in the patterns of cancer mortality. For the age group 15–64 years, the ranges were 1.18 (95% CI: 0.78–1.71) −8.71 (95% CI: 7.18–10.47), *p* < 0.0001 in men and 1.35 (95% CI: 0.92–1.92) −10.83 (95% CI: 8.84–13.14), *p* < 0.0001 in women in 2016. There were higher PMRs among women in the Western Cape, Northern Cape, North West, and Gauteng compared to other areas. Similar patterns were also observed among men in these provinces, except in North West and Gauteng.

**Conclusion:**

The identification of geographical and temporal distributions of cancer mortality provided evidence of periods and districts with similar and divergent patterns. This will contribute to understanding the past, present, future trends and formulating interventions at a local level.

## 1. Introduction

High-quality mortality statistics are crucial for optimal health planning, decision-making, program evaluation, progress monitoring, and resource allocation (1, 2). However, high-quality statistics are often reported at high administrative divisions but not at lower administrative levels for local public health decision-making. There is evidence of disparities in mortality risks at subnational levels driven by age, gender, and social-economic differences (3–5). South Africa (SA) is undergoing an upsurge in non-communicable diseases, such as cancer (6, 7), mainly due to lifestyle-related factors such as obesity, smoking, and alcohol consumption (8). This upsurge is crucial because of its negative impact on economically productive adults (9), who contribute to the economy, and younger and older age groups also depend on them for survival (10). Thus, adult mortality in SA presents a changing set of dynamics challenging the limited healthcare resources of the region (11), because of its impact on the availability and productivity of working adults (12, 13). Mortality atlases often describe the geographical distribution of cancer mortality rates by combining years to make a single period. The burden of diseases, demographic, and many determinants associated with the well-being of populations are dynamic and change over time (14).

Cancer is a rare disease; thus, some geographic areas may present few cases leading to unreliable and unstable results as most atlases use age and gender-adjusted rates or standardized mortality rates (15). In order to provide and understand the patterns of deaths attributed to cancer over a spectrum of relevant subgroups, the time trends and disease mapping for cancer mortality (CM) should be approached in a manner to account for age and sex groups differences at the district level in different years. The best method to understand trends in health indicators is the evaluation of the outcomes of health policies that were implemented in the past and to ascertain the current health status of the population to make a well-informed public health policy in the future (16). Studies in SA investigating time trends and spatial distribution of diseases focused mainly on all causes of mortality combined, not cancer (17, 18). There are studies in SA providing the patterns of cancer incidence, but the reported studies on CM are limited. Furthermore, studies on CM that encompass all cancers and show the burden of CM attributed to various cancers are lacking in SA. To the best of our knowledge, there is no research in SA providing patterns and atlases of the deaths attributable to cancer in age and sex groups per district per year. This study presents age-sex-specific geographical patterns of CM at the district level in SA and their temporal evolutions from 1997 to 2016. This may be crucial for effectively monitoring and evaluating public health policies and programs targeting CM reduction across time and sub-populations.

## 2. Materials and methods

### 2.1. Study population

The mortality data provided by Statistics South Africa (Stats SA) were analyzed using Stata version 17. The registration of deaths in SA is done within three days from the date of the event. A death certificate is issued to the informant after the medical practitioner has prescribed the cause of death. All death notification forms are collected by Stats SA from the Department of Home Affairs in SA. Stats SA is responsible for compiling and processing the death records forms into mortality reports (19). There are many sources of mortality data in SA (20), but the vital death registration data provide the best and most reliable mortality data due to very high levels of completeness. We considered cancer deaths and all-cause mortality stratified by age (0–14 (adolescence), 15–64 (working-age population), and 65+ years old (elderly population) and sex (male and female). We used the 10th International Classification of Diseases codes (ICD-10) to classify the causes of death. Cancer deaths (ICD-10 codes C00-C96) corresponding to various anatomical locations were combined based on six age-sex groupings for each of the 52 districts. The top five leading causes of cancer-related deaths were estimated by adding the absolute numbers of deaths from individual cancers in the twenty-year study period. Finally, the five leading causes of cancer deaths were ranked in descending order.

### 2.2. Statistical analysis

The analyses were performed in two phases. The first phase quantified cancer deaths, computed proportionate mortality ratios (PMRs), and generated corresponding atlases. The second phase focused on identifying the most common cancers and plotting the graphs for PMRs. The PMRs for cancer across the districts for each year were calculated using deaths from cancer and all causes.

$$\mathrm{PMR}=\frac{\begin{array}{c} \mathrm{Number}\;\mathrm{of}\;\mathrm{cancer}\;\mathrm{deaths}\;\;\mathrm{in}\;\;\mathrm{each}\;\;\mathrm{age}\;\\ -\mathrm{sex}\;\mathrm{grouping}\;\;\mathrm{for}\;\mathrm{each}\;\mathrm{year}\; \end{array}}{\begin{array}{c} \mathrm{Number}\;\mathrm{of}\;\mathrm{deaths}\;\;\mathrm{due}\;\;\mathrm{to}\;\;\mathrm{all}\;\;\mathrm{causes}\;\;\mathrm{in}\;\;\mathrm{all}\;\;\\ \mathrm{age}\;\;\mathrm{groups}\;\;\mathrm{in}\;\;\mathrm{the}\;\;\mathrm{same}\;\;\mathrm{sex}\;\;\mathrm{for}\;\;\mathrm{each}\;\;\mathrm{year}\; \end{array}}\times 100$$

The 95% confidence intervals (95% CIs) were calculated based on the Poisson distribution. For the spatial distribution of CM, the data were aggregated at the district level for SA in 1997, 2004, and 2016. The PMRs for cancer per district for three selected years were computed using the cancer deaths in each age-sex grouping per district per year as a numerator, while the number of deaths due to all causes in all age groups in the same sex per district per year served as a denominator. These three years were selected because we wanted to compare the spatial distribution of CM in the era before and after antiretroviral therapy (ART). Hence, ART was initiated in SA around 2004 (21). The shapefiles were joined to their district-specific corresponding aggregated dataset in ArcGIS (22), and the atlases showing the distribution of CM were plotted. Spatial analyses were conducted through Moran's I test. To compute the number of deaths for the most common cancers, the corresponding total number of deaths related to the five major cancers in each age-sex category in that particular year served as a numerator, whereas the number of deaths due to all cancers in all ages in the same sex in that particular year was used as a denominator. These were reported per 100 residents.

## 3. Results

### 3.1. Number of deaths

In males, the numbers of cancer deaths were lowest in the 0–14-year-olds and highest in the 15–64-year-olds in 1997 and other years (Table 1). This pattern was similar among females, although the lowest number of cancer deaths was observed in 2003. The annual number of cancer deaths for males aged 0–14 years was 142 in 1997, while for the 15–64 and 65+ age groups, the numbers were 7,782 and 6,728. For females, the numbers of cancer deaths for 0–14, 15–64, and 65+ years were 141, 6,658, and 5,346 in 1997. The numbers of notified and registered cancer deaths increased sharply from 1997 to 1999 in the working-age and elderly populations. In males, the numbers of cancer deaths for 15–64 and 65+ years were 8,370 and 7,163 in 1999. In 1999, the cases for the age groups 15–64 and 65+ years among females were 7,582 and 6,076. The observed cancer deaths in 2004 were higher than in 1997 in the populace. The annual numbers of cancer deaths among males aged 0–14, 15–64, and 65+ years were 215, 9,177, and 7,753 in 2004. In females, the numbers of cancer deaths for 0–14, 15–64, and 65+ years were 159, 8,953, and 6,853 in 2004. The numbers continued to increase even from 2004 to 2016 in all age and sex groups, although they declined slightly during specific periods. In 2016, the cases for the age groups 0–14, 15–64, and 65+ years were 224, 10,184, and 9,947 among males, whereas they were 180, 11,373, and 9,188 in females.

**Table 1:** Distribution of deaths stratified by sex and age groups across the districts in South Africa from 1997 to 2016.

Year	Cancer deaths						Deaths from all causes	
	0–14 age group		15–64 age group		≥65 age group		All age groups	
	Males	Females	Males	Females	Males	Females	Males	Females
1997	142	141	7,782	6,658	6,728	5,346	177,076	140,011
1998	182	144	8,138	7,239	6,914	5,696	201,954	165,182
1999	190	128	8,370	7,582	7,163	6,076	211,107	178,406
2000	161	133	8,440	7,803	7,117	6,081	224,850	200,673
2001	163	141	8,574	7,987	7,128	6,228	243,934	222,466
2002	184	133	8,723	8,435	7,315	6,330	263,692	248,499
2003	144	125	9,020	8,689	7,426	6,664	290,634	277,119
2004	215	159	9,177	8,953	7,753	6,853	298,660	290,062
2005	175	148	9,008	9,206	7,643	7,085	306,654	301,082
2006	170	126	8,903	9,147	7,776	7,113	314,778	305,591
2007	166	126	9,025	9,172	7,950	7,289	313,555	300,204
2008	160	146	8,924	9,200	7,908	7,241	311,204	295,632
2009	187	164	9,228	9,687	8,123	7,553	304,332	286,096
2010	183	174	9,198	9,669	8,261	7,644	286,717	269,700
2011	178	145	8,953	9,685	8,242	7,731	260,356	242,927
2012	197	154	9,466	10,135	8,668	7,820	253,684	232,094
2013	200	154	9,652	10,369	9,027	8,326	250,224	227,162
2014	204	162	9,821	11,056	9,401	8,728	247,852	224,837
2015	222	186	10,333	11,459	10,003	9,213	253,518	227,634
2016	224	180	10,184	11,373	9,947	9,188	244,942	219,221
Total	3,647	2,969	180,919	183,504	160,493	144,205	5,259,723	4,854,598

### 3.2. All-cancer mortality patterns

**Figure 1:**
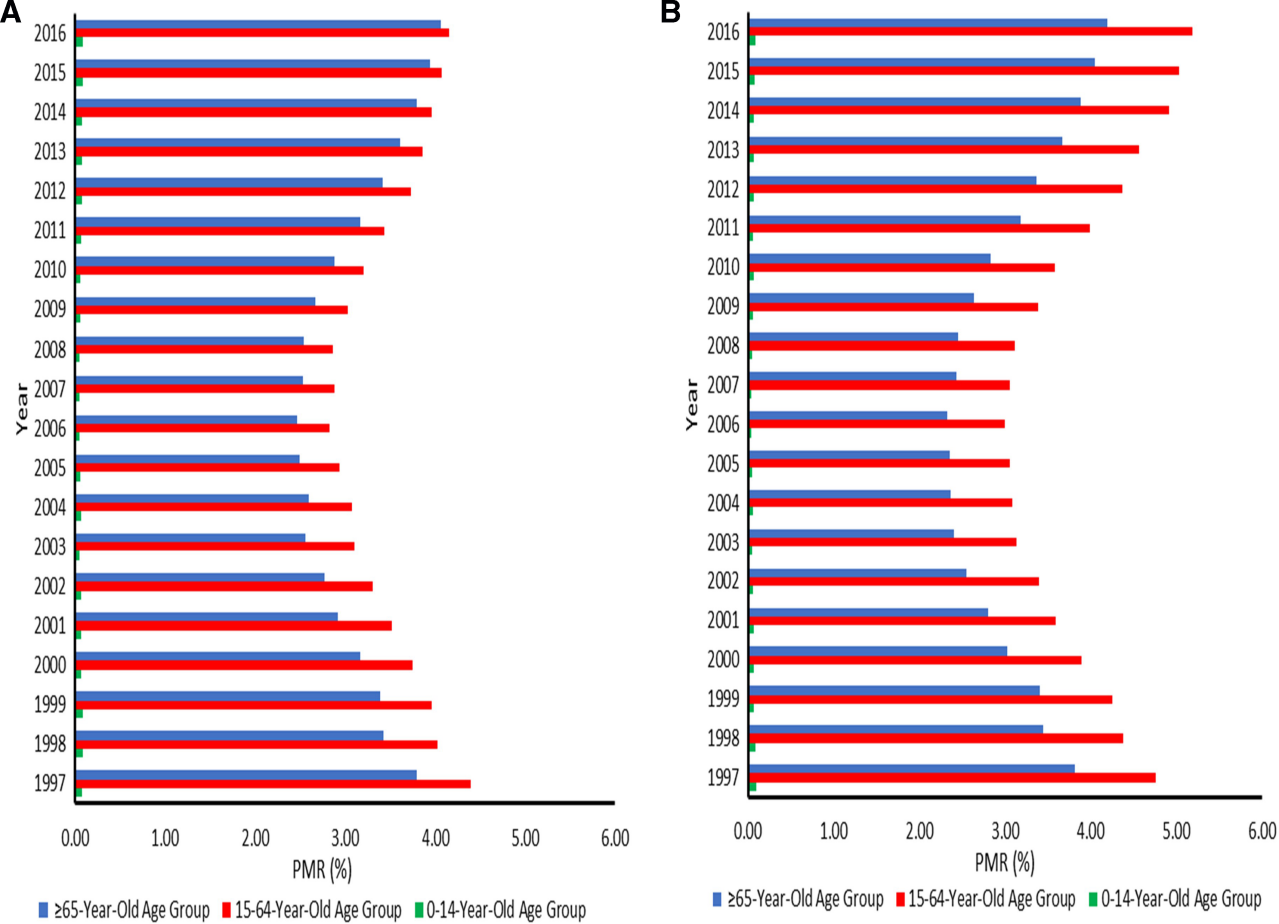
(**A**) Proportionate mortality ratios for cancer among males aged 0–14, 15–64, and 65 years or over across the districts in South Africa from 1997–2016. (**B**) Proportionate mortality ratios for cancer among females aged 0–14, 15–64, and 65 years or over across the districts in South Africa from 1997–2016.

Disparities in the proportion of cancer deaths were observed, with PMRs being lowest in the 0–14 age group, followed by the 65+ age group, and highest in the 15–64 age group, regardless of sex. The PMRs for cancer in the 0–14-year-olds were relatively stable. The PMRs declined from 1997 to about 2006 in the working-age and elderly populations. In men aged 15–64 years, the PMRs increased to levels almost similar to the 1997 levels in 2016. Overall, the PMRs for cancer in the elderly population and women aged 15–64 years increased from 2006 to 2016. This pattern reflects an increasing threat of cancer in SA. Ranges were 2.83 (95% CI: 2.77–2.89) −4.16 (95% CI: 4.08–4.24) among men aged 15–64 years and 2.99 (95% CI: 2.93–3.06) −5.19 (95% CI: 5.09–5.28) among women in this age group. The ranges of PMRs in elderly men and women were 2.47 (95% CI: 2.42–2.53) −4.06 (95% CI: 3.98–4.14) and 2.33 (95% CI: 2.27–2.38) −4.19 (95% CI: 4.11–4.28), respectively (Figures 1A, B).

### 3.3. Study area

**Figure 2:**
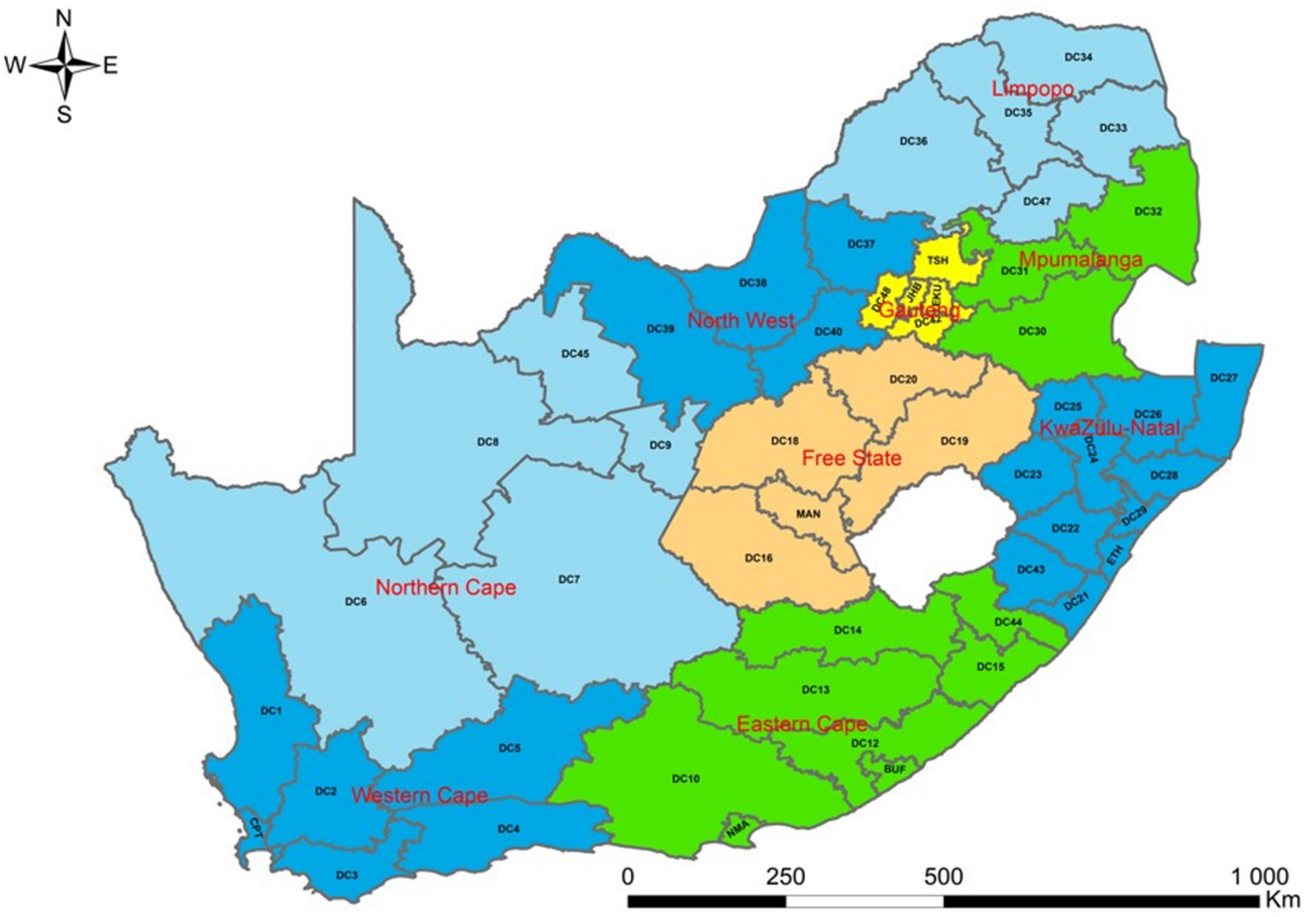
Provinces and districts of South Africa.

SA has nine provinces. The present study was conducted at the district level. However, nine provinces were coded on a map to ensure readability. These regions are further subdivided into 52 districts. There are 44 districts municipalities, and 8 metropolitan districts in SA. Some provinces have many districts, while other regions have few of them. The province with the most districts is KwaZulu-Natal which has 11 districts, followed by Eastern Cape with 8 districts, and 6 districts in Western Cape. There are 5 districts in Free State, Gauteng, Limpopo, and Northern Cape, whereas North West has 4 districts and 3 in Mpumalanga. Of the eight metropolitan districts, three metropolitan districts (City of Ekurhuleni Metropolitan, City of Johannesburg Metropolitan, and City of Tshwane Metropolitan Municipality) are located in Gauteng, two (Buffalo City Metropolitan and Nelson Mandela Bay Metropolitan Municipality) in Eastern Cape, one (City of Cape Town Metropolitan Municipality) in Western Cape, one (eThekwini Metropolitan Municipality) in KwaZulu-Natal and one (Mangaung Metropolitan Municipality) in Free State. The 52 districts in SA are disaggregated into 205 municipalities. The population of SA is estimated to be 60.6 million, and 51.1% of the population is female (23). Gauteng comprises the largest share of the population in SA (26.6%), followed by KwaZulu-Natal (19.0%), and Northern Cape contributes the smallest share of the population (2.2%). In terms of age structure, the populations aged younger than 15 years and 60 years or older are about 28.1% and 9.2% (23) (Figure 2).

### 3.4. Geographical distribution of all-cancer mortality

**Figure 3:**
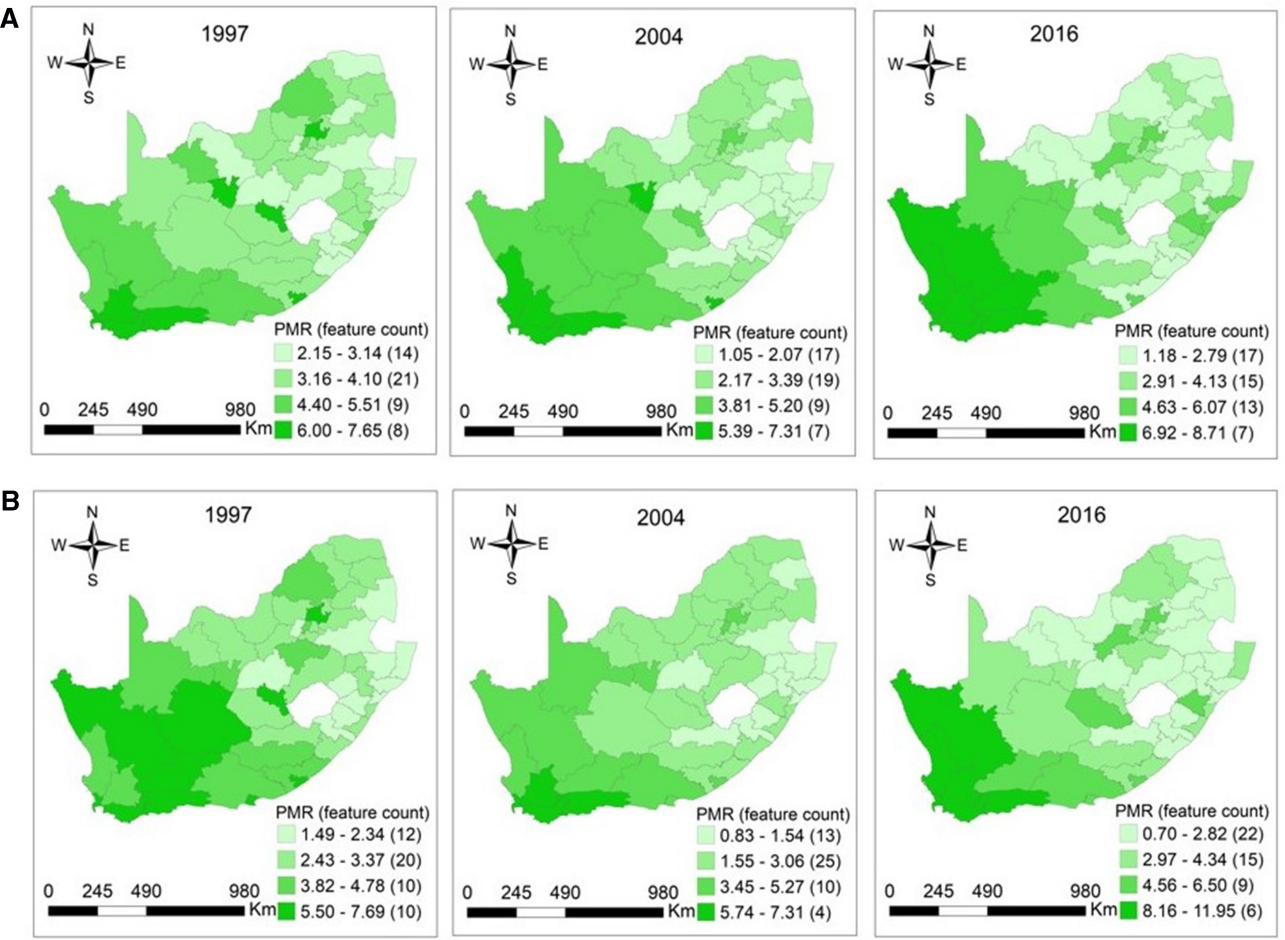
(**A**) Maps of proportionate mortality ratios for cancer among men aged 15–64 years per district in South Africa in 1997 (*p* = 0.0010), 2004 (*p* < 0.0001), and 2016 (*p* < 0.0001). (**B**) Maps of proportionate mortality ratios for cancer among men aged 65 years or over per district in South Africa in 1997 (*p* < 0.0001), 2004 (*p* < 0.0001), and 2016 (*p* < 0.0001).

Cancer mortality is high among individuals with high ages. Furthermore, the five leading causes of cancer death are not childhood cancers. This aspect was reflected by a small number of cancer deaths among 0–14-year-olds in our study. Thus, it is within this context that the maps showing the distribution of CM in this age category were not plotted. There were considerable geographical variations and similarities in the patterns of CM. High cancer mortality occurred in Northern Cape, Western Cape, Eastern Cape, Free State, and Gauteng regions among elderly men in 1997, with PMRs ranging from 1.49 (95% CI: 0.97–2.20) −7.69 (95% CI: 5.39–10.65), *p* < 0.0001. Similar cancer mortality patterns were also observed in men aged 15–64 years in these provinces in the same year, and the reported PMRs were 2.15 (95% CI: 1.51–2.98) −7.65 (95% CI: 7.14–8.18), *p* = 0.0010. In 2004, the magnitude of the highest PMRs which was 7.31 (95% CI: 5.77–9.13), *p* < 0.0001 was similar among men aged 15–64 and 65+ years. The CM in men aged 15–64 years dominated in Western Cape, Northern Cape, and Eastern Cape in 2004, whereas elderly men experienced high CM in Western Cape. Overall, the higher PMRs among men aged 15–64 and 65+ years were observed mainly in Northern Cape and Western Cape regions in 2016. For the working-age and elderly populations, the ranges were 1.18 (95% CI: 0.78–1.71) −8.71 (95% CI: 7.18–10.47), *p* < 0.0001 and 0.70 (95% CI: 0.40–1.13) −11.95 (95% CI: 9.41–14.96), *p* < 0.0001 among men in 2016 (Figures 3A, B).

**Figure 4:**
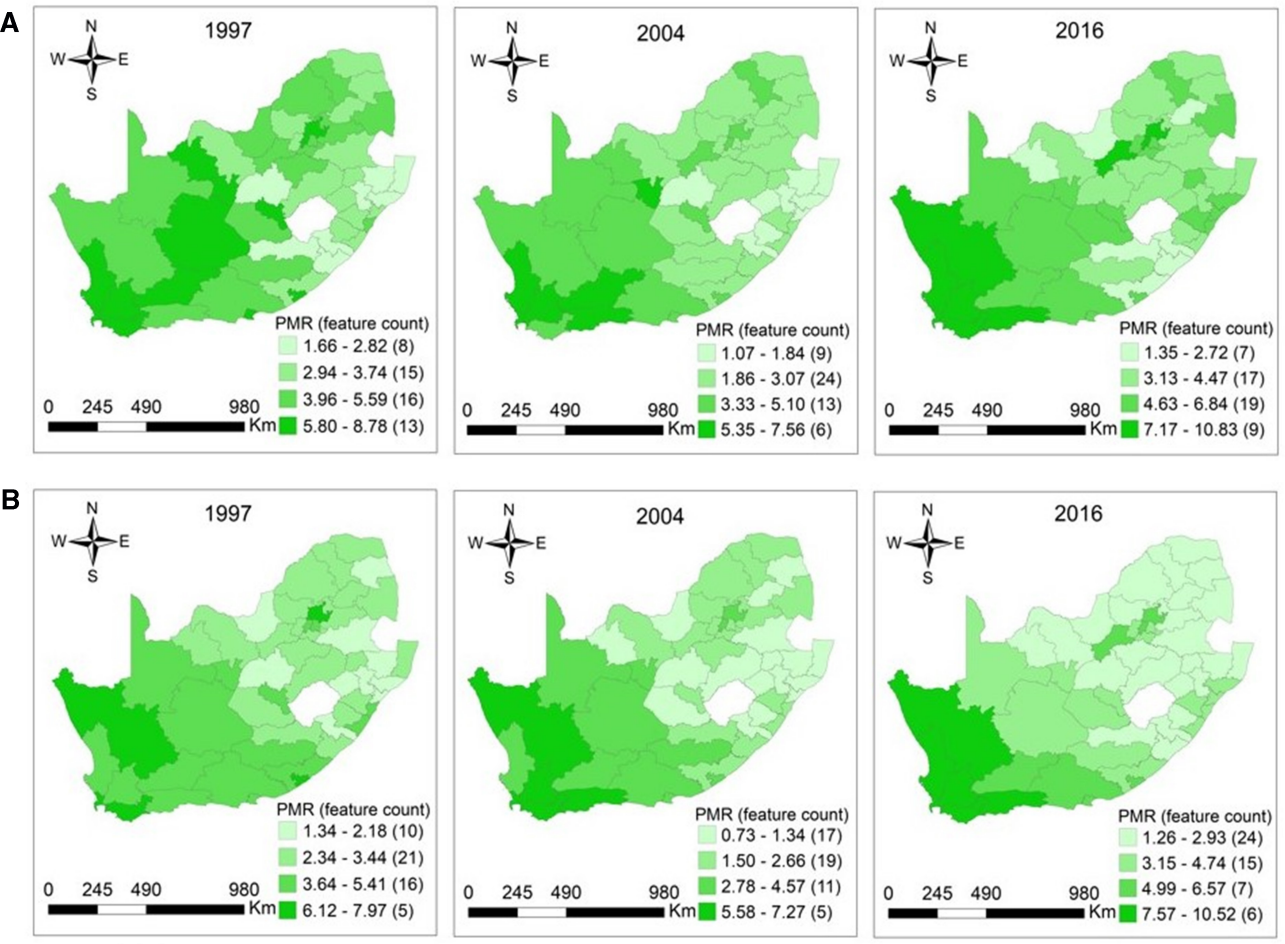
(**A**) Maps of proportionate mortality ratios for cancer among women aged 15–64 years per district in South Africa in 1997 (*p* = 0.0001), 2004 (*p* < 0.0001), and 2016 (*p* < 0.0001). (**B**) Maps of proportionate mortality ratios for cancer among women aged 65 years or over per district in South Africa in 1997 (*p* < 0.0001), 2004 (*p* < 0.0001), and 2016 (*p* < 0.0001).

The distribution of CM among women aged 15–64 was more concentrated in Northern Cape, Western Cape, Eastern Cape, Free State, and Gauteng provinces in 1997. In elderly women, clusters of high CM were located in these provinces in exception of the Free State. In 1997, the reported PMRs among women aged 15–64 and 65+ years ranged from 1.66 (95% CI: 1.00–2.59) −8.78 (95% CI: 8.16–9.43), *p* = 0.0001 and 1.34 (95% CI: 0.95–1.83) −7.97 (95% CI: 7.38–8.60), *p* < 0.0001. In 2004, high cancer mortality occurred in Western Cape and Northern Cape among women aged 15–64 and 65+ years. For the working-age and elderly populations, the observed PMRs were 1.07 (95% CI: 0.75–1.49) −7.56 (95% CI: 7.08–8.06), *p* < 0.0001 and 0.73 (95% CI: 0.50–1.05) −7.27 (95% CI: 5.56–9.34), *p* < 0.0001 in 2004. The distribution of higher CM among women aged 15–64 years was observed in the Northern Cape, Western Cape, North West, and Gauteng regions in 2016, with PMRs ranging from 1.35 (95% CI: 0.92–1.92) to 10.83 (95% CI: 8.84–13.14), *p *< 0.0001. The high CM among elderly women were observed mainly in Northern Cape and Western Cape regions in 2016. The PMRs among elderly women were 1.26 (95% CI: 0.85–1.82) −10.52 (95% CI: 8.56–12.79), *p* < 0.0001 in 2016, respectively (Figures 4A, B).

### 3.5. The five leading causes of cancer death

**Figure 5:**
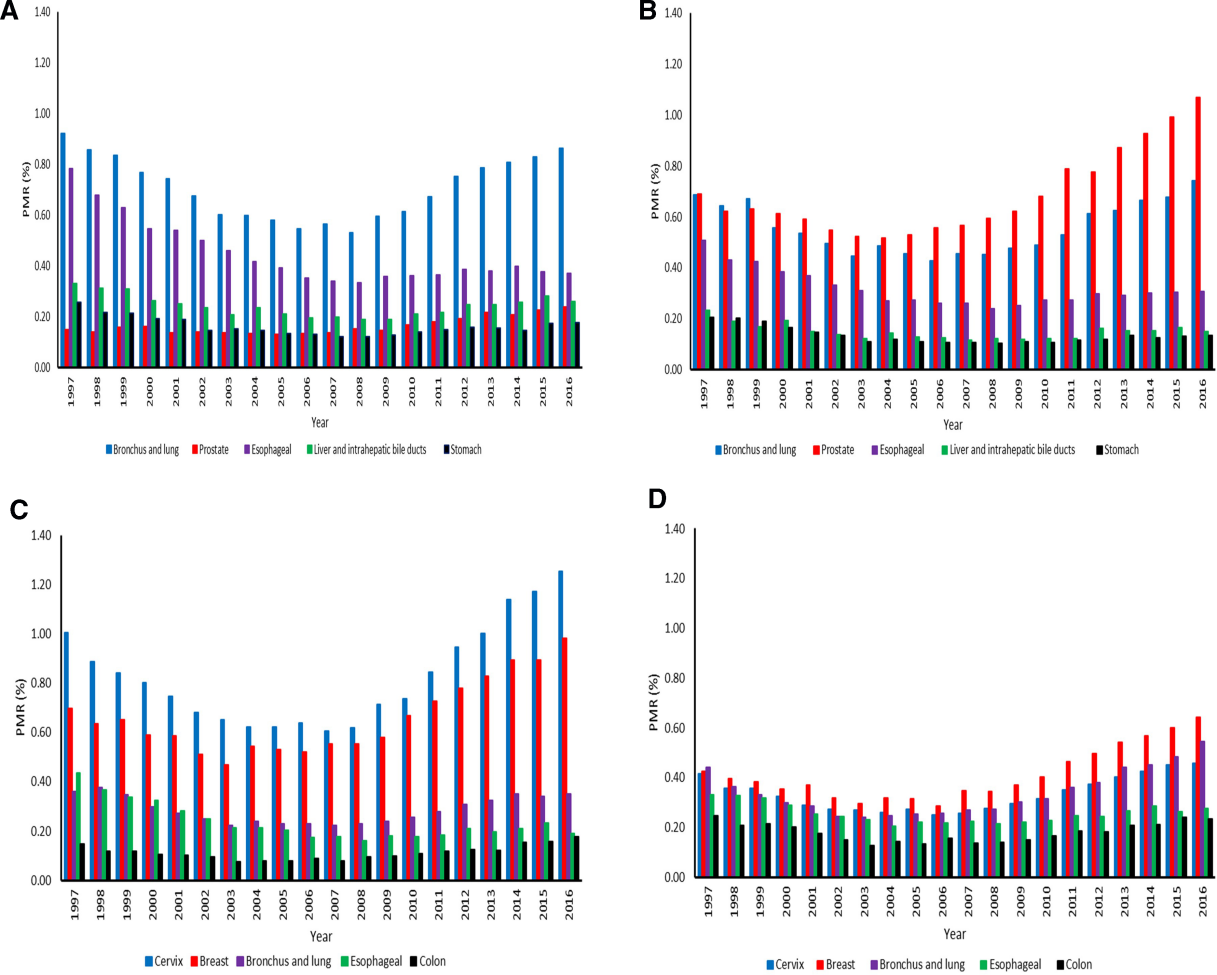
(**A**) Proportionate mortality ratios for top five cancers among men aged 15–64 years across the districts in South Africa from 1997–2016. (**B**) Proportionate mortality ratios for top five cancers among men aged 65 years or over across the districts in South Africa from 1997–2016. (**C**) Proportionate mortality ratios for top five cancers among women aged 15–64 years across the districts in South Africa from 1997–2016. (**D**) Proportionate mortality ratios for top five cancers among women aged 65 years or over across the districts in South Africa from 1997–2016.

Bronchus and lung cancer (PMR = 0.53 (95% CI: 0.51–0.56) −0.92 (95% CI: 0.88–0.97) was the leading cause of cancer death in the entire study period among men aged 15–64 years (Figure 5A). It was followed by cancer of the esophagus; the PMRs were 0.33 (95% CI: 0.31–0.36) −0.78 (95% CI: 0.74–0.83). The PMRs for cancers of the prostate, liver and intrahepatic bile ducts, and stomach were relatively stable in this age group. Among elderly men, prostate cancer (PMRs = 0.52 (95% CI: 0.49–0.54) −1.07 (95% CI: 1.03–1.11) was the leading cause of cancer death, followed by bronchus and lung cancer (PMRs = 0.43 (95% CI: 0.41–0.45) −0.74 (95% CI: 0.71–0.78), whereas esophageal cancer ranked third (PMRs = 0.24 (95% CI: 0.22–0.26) −0.51 (95% CI: 0.48–0.54). Deaths due to liver and stomach cancers were relatively stable over time in men aged 65+ years (Figure 5B). The PMRs for cancers of the cervix, breast, and bronchus and lung among women increased from 2004 to 2016 (Figure 5C). For females aged 15–64 years, cervix cancer (PMRs = 0.62 (95% CI: 0.60–0.65) −1.25 (95% CI: 1.21–1.30) had the highest proportion, followed by breast cancer (PMRs = 0.54 (95% CI: 0.52–0.57) −0.98 (95% CI: 0.94–1.02) in the twenty-year study period. There was a significant decline in esophageal CM (PMRs = 0.16 (95% CI: 0.15–0.18) −0.43 (95% CI: 0.40–0.47) among women from 1997 to 2016. For elderly females, breast cancer (PMRs = 0.29 (95% CI: 0.27–0.31) −0.64 (95% CI: 0.61–0.68) was the leading cause of cancer death from 1998 to 2016. However, bronchus and lung CM (PMRs = 0.24 (95% CI: 0.22–0.26) −0.55 (95% CI: 0.51–0.58) ranked first in 1997, followed by breast cancer. Colon cancer (PMRs = 0.13 (95% CI: 0.11–0.14)−0.25 (95% CI: 0.22–0.27) was the least cause of cancer deaths in the entire study period (Figure 5D).

### 3.6. Contribution of the top five leading causes of cancer-related death

**Figure 6:**
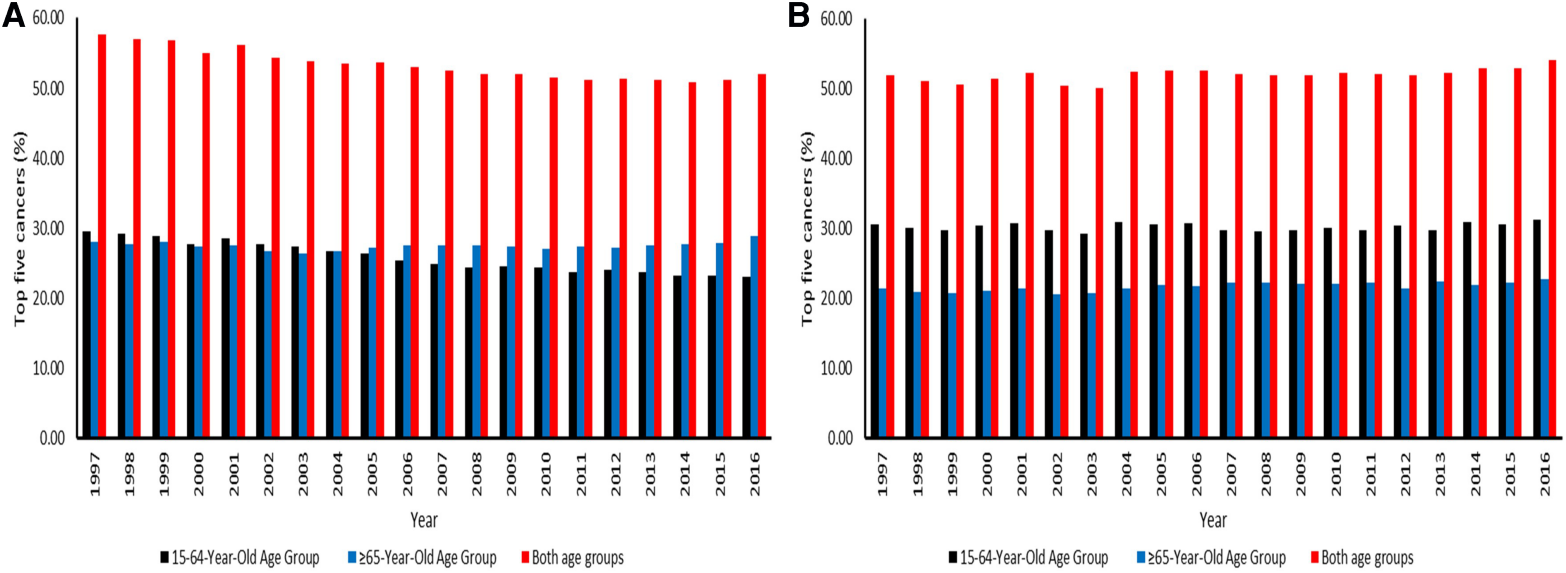
(**A**) Contribution of the top five leading causes of cancer-related death among men aged 15–64 and 65 years or over across the districts in South Africa from 1997–2016. (**B**) Contribution of the top five leading causes of cancer-related death among women aged 15–64 and 65 years or over across the districts in South Africa from 1997–2016.

The mortality for the top five major cancers was higher in men aged 15–64 years than the elderly men from 1997 to 2003. (Figure 6A). Ranges were 27.32%–29.50% among men aged 15–64 years. As time progressed, the elderly population had higher CM than the working-age population from 2005 to 2016. The deaths related to five common cancers in the elderly population were 26.46%–28.94%. The five leading causes of cancer deaths accounted for more than 50% of cancer deaths in both age groups in the entire study period. Ranges were 50.90%–57.60%, with the highest CM in 1997. For the working-age and elderly populations combined among men, the number of deaths attributed to the top five cancers declined from 1997 to 2016. In women, the working-age population had higher CM than the elderly population from 1997 to 2016. The deaths related to the five major cancers in the working-age and elderly populations were 29.25%–31.24% and 20.52%–22.78%. In females, there was an increase in mortality for the top five major cancers from 1997 to 2016 in both age groups. The observed ranges were 50.08%–54.01%, as the highest mortality was in 2016 (Figure 6B).

## 4. Discussion

This study was undertaken to provide patterns and spatial distributions of CM to better understand the evolution of age-sex PMRs for cancer over time and space in SA. The vital death statistics data have been of high coverage, especially at the level needed for assessing patterns and mapping. The study findings will contribute towards a reflection on the past, present, and future trends of CM in SA. We are aware that this study did not include the calculation of CM rates, which may estimate the risks of this disease. Most mid-year population estimates in SA are at the regional level and not often given by age-sex groups at the district level. The adopted approach has allowed for the computation of PMRs for cancer by six age-sex groups and the identification of districts presenting high and low CM. The numbers of cancer deaths increased from 1997 to 2004 in all age and sex groups. The increase may be indicative of population growth and better reporting coverage. The numbers of notified and registered cancer deaths continued to increase even from 2004 to 2016, although they declined slightly during specific periods. This may be due to variations in cancer deaths recorded across the districts in SA, which may suggest the variation in the quality and completeness of mortality data. We found disparities in all-cancer mortality in the age-sex groups, with the lowest proportions in children, followed by the elderly population, and the highest in the working-age population. The low CM in the 0–14 year-olds could be explained by the fact that pediatric cancer is underreported in SA. There are about 50% of childhood cancer cases that are not diagnosed in SA (24). The stability of the trend among children in SA probably is due to the lower incidence rate of childhood cancers, even far lower than in developed countries such as the United States. A study done in SA reported the age-standardized incidence rate (ASR) of 45.7 per million children (25), whereas the ASR in high-income countries was 180 per million (26). A study done in Nigeria reported that hematological malignancies accounted for most cancer deaths among children (27). This present study did not assess the pattern of deaths due to hematological malignancies because of the criteria used to select the five leading types of cancer death. However, hematological malignancies were included when the pattern of deaths attributable to all cancers was assessed.

Age is positively correlated with cancer (28). This could explain why cancer contributed to more deaths in the working-age and elderly populations than in children. The improvement of life expectancy in SA after the ART era could be the contributory factor to the high CM in these age categories. Of notable importance, cancer contributed to more deaths among individuals aged 15–64 years than 65+ years. The predominance of cancer deaths among working-age individuals may be due to either age profile of the population or actual disparities in CM rates in SA. The lower CM among the elderly population than working-age individuals signifies that the patients do not live long after they were diagnosed with cancer or cancer cases were successfully treated. Furthermore, there is a long waiting period from screening to initiation of treatment amongst cancer patients in SA. The waiting times for radiation treatment of gynecological and prostate cancers in SA take many weeks. Delaying cancer treatment for more than 12 weeks may be harmful. This delay is caused by the lack of access to care linked with socio-economic status, race, insurance status and urban-rural location, which significantly affect vulnerable groups, namely black Africans, poor, uninsured, and rural residents (19). Another possible explanation is that the patients might have started to seek care from traditional healers before going to cancer centers due to stigma and lack of awareness. This resulted in the presentation of cancer cases at the advanced stage; hence it is often difficult to cure cancer at its advanced stage. More worrisome is the observed increase of CM from 2006 to 2016 in the population aged 15–64 and 65+ years, which calls for the implementation of necessary interventions. The lack of awareness among the general public and healthcare professionals of cancer negatively impacts the number of patients diagnosed and those referred to appropriate services; hence, patients may die from this disease. The avoidance of these cancer deaths may include strengthening awareness, diagnostic capacities, and early treatment (29). The spatial distributions of CM showed differences between districts, indicating disparities in access to health care quality, age distribution, and the burden of cancer disease. Cancer services such as diagnostic, curative, rehabilitative, psycho-social or palliative services in SA are more concentrated in two provinces, namely, Western Cape and Gauteng. Despite the availability of cancer services in the Western Cape and Gauteng provinces, deaths due to cancer predominated in those provinces in 1997. This may be explained by the movement of patients diagnosed with cancer from their under-resourced provinces to the Western Cape and Gauteng to seek care. This increases the number of cancer patients in the well-resourced provinces leading to inadequate staffing ratio, low survival rate, and poor quality of survivorship. This study found the lowest PMR among elderly women in 2006. This suggests that women live longer than men; hence men are highly likely to involve themselves in risky behavioral patterns such as heavy drinking (30), which may contribute to cancer morbidity and mortality. However, women in this age group may die from other conditions that are linked with aging. SA is experiencing demographic, epidemiological, and nutritional transitions contributing to lifestyle-related diseases and mortality, such as cancer. Men and women aged 15–64 and 65+ years are crucial in studying CM and other non-communicable diseases, considering that these age groups are more likely to develop and succumb to these diseases. In addition to the health impact, the findings from this study have some important economic implications.

The highest mortality was observed in the 15–64-year-olds, the economically active, contributing to the country's economy. In SA, economically reproductive adults are also helpful in the welfare of younger and older age groups. In males and females aged 15–64 years, the high CM may negatively impact the economy. Cancer constituted the majority of deaths in the Western Cape, Northern Cape, North West, and Gauteng in women aged 15–64 years. CM also predominated among men in these provinces, except in North West and Gauteng. The CM preponderance in Western Cape and Gauteng provinces could be due to the high rate of inter-provincial migration among potential job-seekers and employees. Gauteng and Western Cape dominate the economy in SA; hence these provinces are zones of attraction. The in-migrants modify their lifestyles as they move from their places of origin to urbanized places and adapt to urbanization associated with cancer (31). Therefore, patients diagnosed with cancer may die from this disease if untreated. Given an increased unemployment rate in SA, many families depend on old-age pensions for survival; hence the high CM among the 65+ age group may increase poverty in many families. There are over three-quarters of African adults age-eligible for a pension who stay with at least one individual younger than 21 years, and the majority live in households containing three or more generations (32).

Thus, the findings presented in this study will assist policymakers by providing empirical evidence to help them make and map policies to narrow the differences in access to quality healthcare services for districts. However, some of these initiatives were implemented, as detailed within the National Cancer Strategic Framework (33). These initiatives aimed to reduce and combat communicable and non-communicable diseases, increase primary health access to families and communities, and achieve universal health care coverage. Overall, the five leading causes of cancer death were similar among the working-age and elderly populations, but some differences were based on ranking and sex. Similar cancer profiles with some insignificant variations in their relative rankings have been reported in SA (7, 34, 35). Lung and esophageal cancers were among the five leading causes of cancer death in both sexes. The high prevalence of smoking in SA could be responsible for lung and esophageal CM patterns in the populace. Hence, smoking is a common risk factor for lung and esophageal cancers (36). The estimated prevalence of smoking in adult men and women in SA is 26.5% and 5.5% (37). Among men, the major contributors to CM in the working-age category were lung and esophageal malignancies, whereas lung and prostate cancers were the leading causes of cancer death in the 65+ age group. Advanced age is associated with prostate cancer (38); this explains the pattern of high CM observed among the 65+ age group. Worldwide, lung and prostate cancers are the two most common cancers that affect men, whereas lung and liver malignancies cause more deaths among males (34). Cancers of the cervix and breast caused the greatest mortality in women aged 15–64 years, while the majority of deaths in elderly women were due to breast and lung malignancies. Interestingly, deaths due to cervix cancer among females aged 15–64 hugely declined from 1997 to 2003 and increased from 2004 to 2016. This signifies that HIV-positive women might have died before developing cervical cancer, but the introduction of ART in SA from 2004 to 2016 has increased the life expectancy, which is long enough to enable the development of cervical cancer. There are projections that the incidence of cervical cancer will sharply increase in SA (39). There is high accessibility, affordability, and availability of ultra-processed food in rural and urban areas in SA. The ultra-processed food consumed is associated with breast cancer (40). Among females worldwide, breast and colon cancers are the top two cancers diagnosed, whereas breast and lung cancers are the leading causes of cancer death (34). The high incidence of this disease may increase the number of deaths related to the five major cancers. Hence, the five leading causes of cancer death in our study were among the top ten cancers diagnosed in South African men and women in 2019 (41). Compared to children, the risk factors associated with many cancers are more prevalent in working-age and elderly populations. Therefore, the high prevalence of cancer risk factors among these age groups studied could lead to high CM.

We found that the five leading causes of cancer deaths contributed more than 50% of cancer deaths in both working-age and elderly populations, regardless of sex. The National Department of Health in SA has prioritized the cancers of the lung, colon, cervix, prostate, and breast based on high incidences of these cancers (33). The high incidences of these major cancers were also reported in sub-Saharan Africa (42). Based on the findings of our study, it is suggested that all the top five cancers in each sex should be given priority when formulating the interventions to fight cancer. Furthermore, the implementation of interventions should target both working-age and elderly populations. In South Africa, the numbers of cancers diagnosed among males and females increased from 26,538 to 41,491 and 28,748 to 43,811 between 2008 and 2019 (43). Studies conducted in South Africa from 1998 to 2002, 2003 to 2007, and 2008 to 2012 reported similar cancers diagnosed (44–46), although there were some differences based on ranking and sex. Esophageal cancer (30.5%) was the first most frequent cancer diagnosed, followed by prostate cancer (14.8%), Kaposi sarcoma (7.0%), lung cancer (5.2%), and liver cancer (5.1%) in men from 2008 to 2012, while cervical cancer accounted for 34.5%, esophageal cancer (19.9%), breast cancer (14.4%), Kaposi sarcoma (3.8%), and ovarian cancer (2.8%) in women (46).

Given that cancer treatment is expensive, there should be more financial investment in cancer and educational campaigns at a local level to avoid the diagnosis of this disease at an advanced stage. Investments in cancer research that can produce a return on investment could help drive policy (47). Addressing the inequalities within provinces can yield positive results in the fight against cancer. This can reduce pressure caused by in-migrants seeking care in the cancer centers in other provinces. A comprehensive cancer surveillance system may be crucial for reducing cancer morbidity and mortality in SA (48). It is important to emphasize that the PMRs were used to estimate whether the proportion of deaths due to cancer in residents from specific districts increased or decreased across time. This study aims not to draw conclusions about districts but rather to identify whether these districts serve as a risk marker for CM. This information could necessitate further studies to investigate factors contributing to geographic variation in CM. This study has some strengths and limitations. One of the advantages is to utilize the underutilized database to compute and compare PMRs by six age-sex groups per district per year in SA. The number of cancer deaths alone may not indicate the seriousness of this disease; hence this study used the population totals as the denominator. The advantage of PMRs is that they do not need the usage of the mid-year population as a denominator in computing them. Therefore, there was no interpolation of population totals which could have resulted in incorrectness of the mid-year population totals at the district level. Furthermore, the interpolation of population totals could have affected the computation and interpretation of some PMRs, especially for small and sparser districts. The aspect of the study that might be considered as a limitation is the lack of finer age group categories to evaluate cancer mortality, especially in the 40–64 age groups. Another limitation is that PMRs are sensitive to changes in detection or diagnostics and may not accurately reflect cancer risks.

## 5. Conclusions

This study has shown the patterns and geographical distribution of CM for different sex and age groups at the district level. This adopted approach has helped identify common and different patterns in geographical and temporal distributions of PMRs for cancer in SA. This may give evidence of periods and areas presenting high or low CM and, therefore, will significantly contribute to understanding the past, present, and future trends of CM in different areas to formulate a more focused policy at a local level. The identification of districts presenting high or low CM would be an interesting area for further research to elucidate the factors driving these patterns.

## Data Availability

The original contributions presented in the study are included in the article, further inquiries can be directed to the corresponding author.
